# High sustained virologic response rates, regardless of race or socioeconomic class, in patients treated with chronic hepatitis C in community practice using a specialized pharmacy team

**DOI:** 10.1097/MD.0000000000034183

**Published:** 2023-07-28

**Authors:** Saatchi Kuwelker, Eugenia Tsai, Lily Kuo, Jae Kim, Timothy Van Frank, Robert Mitchell, Ruben Ramirez, Richard Guerrero, Bryan Hanysak, Carmen Landaverde, Fabian Rodas, Eric Lawitz, Tamneet Basra, Harry Nguyen, Kim Christensen, Clarissa Vaughn, Kim Hinojosa, Nina Olvera, Edna Caraballo-Gonzalez, Emma Pham, Lisa D. Pedicone, Fred Poordad

**Affiliations:** a University of Texas Health, San Antonio, TX; b Texas Liver Institute, San Antonio, TX; c Corpus Christi Gastroenterology, Corpus Christi, TX; d Providence Gastroenterology and Liver Associates, El Paso, TX; e Sun City Gastroenterology and Hepatology, El Paso, TX; f Waco Gastroenterology Associates, Waco, TX; g Health Outcomes Centers, San Antonio, TX; h R&R Strategies, Inc., Bedminster, NJ.

**Keywords:** HCV DAAs, HCV disparities, hepatitis C, SVR, SVR by race, SVR by socioeconomic class

## Abstract

Approved direct-acting antiviral (DAA) regimens against hepatitis C virus (HCV) can cure nearly all patients; however, socioeconomic disparities may impact access and outcome. This study assesses socioeconomic factors, differences in insurance coverage and the drug prior authorization process in HCV-infected patients managed in community practices partnered with a dedicated pharmacy team with expertise in liver disease. This Institutional Review Board-approved, ongoing study captures data on a cohort of 2480 patients from community practices. Patients had chronic hepatitis C and were treated with DAA regimens selected by their physician. The HCV Health Outcomes Centers Network provides comprehensive patient management including a dedicated pharmacy support team with expertise in the prior authorization process. In this cohort, 60.1% were male, 49% were Hispanic Whites (HW), 37% were Non-Hispanic Whites (NHW), and 14% were Black/African American (BAA). Eighty-seven percent of patients were treatment-naïve, 74% were infected with genotype 1 virus and 63% had advanced fibrosis/cirrhosis (F3/F4 = 68.2% HW, 65.6% BAA, 55.4% NHW). Forty percent of patients were on disability with the highest percentage in the BAA group and less than one-third were employed full time, regardless of race/ethnicity. Medicare covered 42% of BAA patients versus 32% of HW and NHW. The vast majority of HW (80%) and BAA (75%) had a median income below the median income of Texas residents. Additionally, 75% of HW and 71% of BAA had median income below the poverty level in Texas. Despite the above socioeconomic factors, 92% of all prior authorizations were approved upon first submission and patients received DAAs an average of 17 days from prescription. DAA therapy resulted in cure in 95.3% of patients (sustained virologic response = 94.8% HW, 94.0% BAA, 96.5% NHW). Despite having more advanced diseases and more negative socioeconomic factors, >94% of HW and BAA patients were cured. Continued patient education and communication with the healthcare team can lead to high adherence and > 94% HCV cure rates regardless of race/ethnicity or underlying socioeconomic factors in the community setting.

## 1. Introduction

There are an estimated 2.4 million people currently living with hepatitis C virus (HCV) infection in the USA, making it the most prevalent chronic viral infection in the USA.^[1]^ Despite advances in detection and curative treatment, the disease burden from the virus continues to increase. In response, the World Health Assembly announced a global strategy in 2016 to reduce infections by 90% and deaths by 65% worldwide by urging countries to implement policies to meet this demand.^[2]^

The United States Preventive Services Task Force published guidelines in 2020 recommending wider HCV screening for all patients between the ages of 18 and 79 regardless of risk group.^[3]^ Prior to these new recommendations, it is estimated that in patients inflicted with HCV infection, approximately 50% had HCV testing that confirmed infection, however only 16% were treated with antiviral medications and only 9% of patients living with HCV infection successfully achieved sustained virologic response (SVR).^[4]^ These estimates highlight the system failure in successful identification and availability of curative treatment for HCV patients.^[5]^ There is concern that this breakdown disproportionately affects racial and ethnic minorities in the USA, as there is well-established evidence of suboptimal access to healthcare in this population. Of patients with HCV infections, the prevalence is greatest in non-Hispanic blacks with twice the reported infection rate compared to non-Hispanic White (NHW).^[5]^

This disparity amongst HCV-infected individuals can be particularly evident in racial and ethnic minorities when left untreated. The disease progression in untreated HCV infection can be severe and irreversible, progressing from infection to liver fibrosis, cirrhosis, hepatocellular carcinoma (HCC), and eventually liver failure and death. The disease burden of untreated HCV contributes to rising healthcare costs, as patients often seek medical attention for the first time when physical manifestations of liver disease arise, at which time the disease is usually incurable and associated with higher morbidity and mortality. Direct-acting antiviral (DAA) agents can be curative but are expensive and often total >$70,000 for a full course of treatment.^[6]^ To improve patient outcomes, it becomes imperative to focus on early identification of disease with wider access to curative and complete treatments.

Several aspects such as the cost of treatment and socioeconomic factors become significant determinants in patient access to testing and treatment. Previous data show a positive correlation between higher income class and DAA use.^[7]^ Though there have been some studies demonstrating the Black-White-Hispanic gap among DAA use observed in 2014 closing by 2016, many studies continue to show racial minorities having lower DAA initiation and treatment rates.^[7–10]^

With the rise in immigration of minority populations in the USA, identifying and understanding demographic data and disease characteristics in this group is imperative to closing the disparity in infection and treatment rates. The aim of this study seeks to understand disease characteristics and demographic data to identify racial and socioeconomic disparities in testing and treatment in a large Texas cohort of patients managed by a specialized team.

## 2. Methods

### 2.1. Patient population

This Institutional Review Board-approved, observational protocol captured SVR and pharmacy data. The analysis was based on a cohort of 2480 patients from community practices who started on therapy between January 2017 and September 2021. Patients were managed by the Health Outcomes Centers (HOC) health care team.

Inclusion criteria included patients ≥18 years of age with chronic hepatitis C (any genotype) who were treated with a DAA regimen selected by the provider. Prescribed treatment selected by the provider ranged from 8 to 24 weeks, was based on FDA approved labeling, and depended on drug regimen, genotype, baseline viral load, and/or presence of cirrhosis.

Insurance carrier, prior authorization process data, and specialty pharmacy data were captured. DAA medications required prior authorization by insurance companies and were shipped by a specialty pharmacy via carrier service to the patient’s home. Medication was shipped as a 4-week supply and a HOC pharmacist oversaw refills and patient compliance.

Median income was calculated based on the patient’s US postal code (5-digit zip code). Zip codes are a series of 5-digit numbers that communicate information, including median income, about people within different geographic groupings. Race was patient-defined.

Liver staging was based on results from FibroTest^TM^ (FibroSURE in the United States) or FibroScan^®^ (Echosens). If a patient had results from both FibroTest^TM^ and FibroScan^®^ with correlation to different fibrosis stages, the higher fibrosis stage was selected (e.g., F4 vs F3). The cutoffs used for FibroTest^TM^ were F0 ≤ 0.21, F1 = 0.22–0.31, F2 = 0.32–0.58, F3 = 0.59–0.72, F4 ≥ 0.73. The cutoffs used for FibroScan^®^ were F0 ≤ 4.5 kPa, F1 = 4.5–6.9 kPa, F2 = 7.0–8.9 kPa, F3 = 9.0–13.9 kPa, F4 ≥ 14 kPa. Standard of care clinical data and pharmacy data were captured in a RedCap Health Insurance Portability and Accountability Act (HIPAA) compliance database.

### 2.2. Patient visits

The HOC team conducted an educational session (in person or via telehealth) at the time of enrollment. Patient education relating to what chronic hepatitis C is, the risk factors associated with infection, the manifestations of liver disease progression if left untreated, and the risk of reinfection, if risky behaviors were resumed after being cured, was provided. Patients also received a handout describing the importance of dosing compliance and how to avoid drug:drug interactions.

At treatment week 4, laboratory tests were done and a telehealth visit was conducted to review progress and ensure drug compliance in all patients. If a patient had detectable HCV ribonucleic acid (RNA) at treatment week 4 or had decompensated cirrhosis, laboratory tests were repeated at treatment week 8 and a telehealth visit was conducted. In patients with decompensated cirrhosis, laboratory tests were performed every 4 weeks while patients were on treatment. All patients had laboratory tests repeated 12 weeks after they completed treatment. A telehealth visit was also conducted at that time to review results and reeducate on the potential for reinfection if exposed to risk factors. Patients with cirrhosis continued to be followed by liver specialists while noncirrhotic patients were managed by their primary care providers.

## 3. Results

### 3.1. Description of the study cohort

A total of 2480 patients were included in the study of which 37% were NHW, 49% Hispanic Whites (HW) and 14% Black/African Americans (BAA) (Table 1). The mean age of all patients at baseline when presenting for treatment was 56 years compared to 61 years in the BAA population. Females included 40% of patients, with similar distribution amongst all 3 racial categories. The mean age of HCV diagnosis was 49 in BAA patients compared to 46 years old in HW and 40 years old in NHW. Sixteen percent of patients used marijuana and 63% were current/past tobacco users. Of all lifetime tobacco users 63% belonged to the NHW class.

**Table 1:** Demographic data by racial class.

Demographic characteristic	Non-Hispanic White (NHW) (N = 916)	Black/African American (BAA) (N = 350)	Hispanic White (HW) (N = 1214)	Total (N = 2480)
No. of patients	916 (37%)	350 (14%)	1214 (49%)	2480 (100%)
Age at baseline, years; mean (range)	55 (18, 91)	61 (24, 83)	54 (18, 87)	56 (18, 91)
Females, n %	407 (44%)	142 (41%)	441 (36%)	990 (40%)
Age diagnosed with HCV, years; mean (range)*	40 (12, 72)	49 (14, 83)	46 (12, 81)	45 (12, 83)
Substance use, n (%)				
Marijuana use	87 (18%)	30 (14%)	99 (14%)	216 (16%)
Tobacco use				
Never used	204 (29%)	94 (32%)	406 (44%)	704 (37%)
Ex-smoker	151 (24%)	66 (23%)	201 (23%)	428 (22%)
Current use	349 (50%)	133 (45%)	303 (33%)	785 (41%)

HCC = hepatitis C virus.

* Missing patient data.

### 3.2. Disease characteristics by racial class

**Figure 1.**
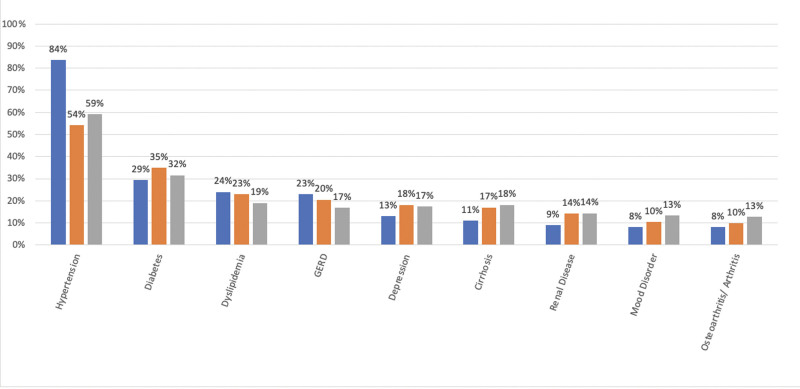
Most common comorbid chronic conditions by racial class. Blue bars = Black/African American, orange bars = Hispanic White, grey bars = non-Hispanic White.

HCV genotypes were similarly distributed across racial classes with 61% of patients infected with genotype (GT) 1a. Very few BAA were infected with GT3 (2%) compared to NHW (14%) and HW (15%). With prior history of HCC, HW had a higher percent (7%) at baseline compared to BAA (2%) and NHW (3%). On the contrary, non-HCC cancer history was higher in NHW and BAA compared to HW. Although 2% of NHW and 3% of HW had a prior history of liver transplant, none of the patients in the BAA group had undergone liver transplant. Four percent (4%) of HW and BAA were dialysis dependent; however, only 1% of NHW were. The most common chronic comorbid condition was hypertension which was reported at much higher rates in BAAs (84%) compared to NHWs (59%) and HWs (54%), Diabetes, the second most common comorbidity, was reported in a higher percentage of HWs (35%) compared to NHWs (32%) and BAAs (29%) (Fig. 1).

Approximately 98% of patients had liver staging done on presentation with 38% having a fibrosis score of F4. A higher percentage of BAA (65%) and HW (68%) had advanced fibrosis/cirrhosis (F3/F4) at the time of treatment compared to NHW (55%). Nearly all patients were treatment-naïve with only 6.2% of patients having previously failed an interferon-based regimen and 7.1% having previously failed a DAA-based treatment. There were no differences between HW, BAA, and NHW (Table 2).

**Table 2:** Disease characteristics by racial class.

Disease characteristic, N (%)	Non-Hispanic White (NHW) (N = 916)	Black/African American (BAA) (N = 350)	Hispanic White (HW) (N = 1214)	Total (N = 2480)
HCV genotype*				
1a	531 (58%)	235 (67%)	731 (61%)	1497 (61%)
1b	91 (10%)	87 (25%)	114 (10%)	292 (12%)
1 (unknown)	7 (1%)	1 (0.3%)	13 (1%)	21 (1%)
2	155 (17%)	12 (3%)	160 (13%)	327 (13%)
3	128 (14%)	7 (2%)	177 (15%)	312 (13%)
4, 5, 6	4 (0.4%)	9 (3%)	9 (1%)	22 (1%)
Prior cancer history				
None	725 (90%)	295 (92%)	894 (89%)	1914 (90%)
Hepatocellular carcinoma	27 (3%)	7 (2%)	67 (7%)	101 (5%)
Other	54 (7%)	20 (6%)	46 (5%)	120 (6%)
Prior liver transplant	11 (2%)	0	23 (3%)	34 (2%)
Dialysis dependent	4 (1%)	11 (4%)	31 (4 %)	46 (3%)
Fibrosis score*				
F0	161 (18%)	39 (12%)	131 (11%)	331 (14%)
F1	143 (16%)	47 (14%)	140 (12%)	330 (14%)
F2	92 (10%)	30 (9%)	103 (9%)	225 (10%)
F3	196 (22%)	102 (30%)	309 (26%)	607 (25%)
F4	295 (33%)	119 (35%)	492 (42%)	906 (38%)
Prior HCV therapy				
Interferon-based	57 (6%)	16 (5%)	78 (7%)	151 (6%)
DAA-based	58 (6%)	23 (7%)	93 (8%)	174 (7%)

DAA = direct-acting antiviral, HCC = hepatitis C virus.

* Missing patient data.

### 3.3. Socioeconomic disparities by racial class

**Figure 2.**
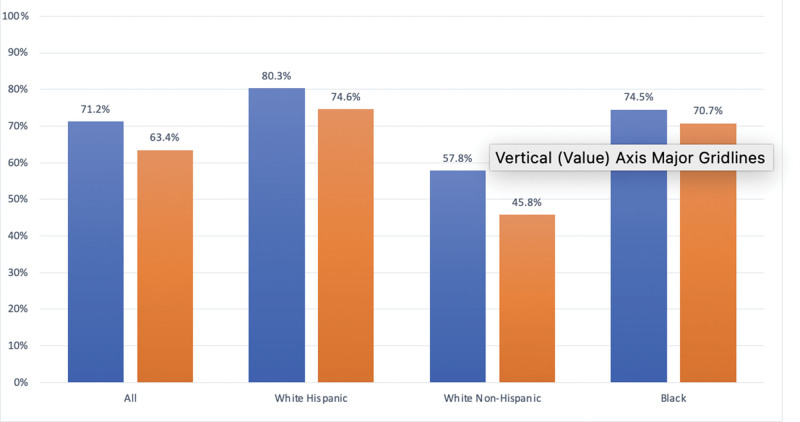
Income distribution by racial class. Blue bars: % of patients with median income below median income of Texas. Orange bars: % patients with income below poverty income of Texas. Orange bars: % patients with income below poverty income of Texas.

Table 3 depicts the distribution of socioeconomic characteristics by racial class. A higher percentage of BAA (47%) and HW (42%) patients were on disability compared to NWH (35%). In the full dataset, 71.2% of patients had a median income below the median income reported for Texas residents. This was driven by HW (80.3%) and BAA (74.5%) and not NHW (57.8%). Additionally, a higher percentage of HW (74.6%) and BAA (70.7%) patients had incomes below the poverty line of the state compared to NHW (45.8%) (Fig. 2). This parallels higher rates of Medicare/Medicaid coverage in the HW and BAA groups.

**Table 3:** Key socioeconomic characteristics by racial class.

Socioeconomic characteristics N (%)	Non-Hispanic White (NHW) (N = 916)	Black/African American (BAA) (N = 350)	Hispanic White (HW) (N = 1214)	Total (N = 2480)
Current employment status*				
Disability	202 (35%)	113 (47%)	315 (42%)	630 (40%)
Full time	183 (32%)	62 (26%)	213 (28%)	458 (29%)
Retired	100 (17%)	46 (19%)	94 (12%)	240 (15%)
Unemployed/not actively seeking work	40 (7%)	10 (1%)	72 (10%)	122 (8%)
Part/time	25 (4%)	3 (1%)	26 (3%)	54 (3%)
Unemployed/unknown if seeking work	16 (3%)	4 (1%)	18 (2%)	38 (2%)
Unemployed/actively seeking work	11 (2%)	0	16 (2%)	27 (2%)
Student	4 (0.7%)	1 (0.4%)	4 (1%)	9 (0.6%)
Insurance				
Private	398 (43%)	99 (28%)	430 (35%)	927 (37%)
Medicare	297 (32%)	105 (42%)	389 (32%)	832 (33%)
Medicaid	221 (24%)	146 (30%)	395 (33%)	721 (29%)
HCV treatment approval				
Approved with first prior auth request	835 (92%)	315 (91%)	1105 (92%)	2255 (92%)
Days to shipment of drug, mean (range)	18 (1, 169)	18 (1, 199)	17 (0, 213)	17 (0, 213)
Income per subgroup, median (range)	$43,108	$32,202	$34,456	$37,477

HCV = hepatitis C virus.

* Missing patient data.

Prior authorization was granted in 92% of patients upon first request, regardless of race. Mean number of days from prescription to delivery of DAA treatment was 18 days which was consistent across all racial groups.

### 3.4. Treatment outcomes by racial class

All patients were treated with DAA regimens as outlined in Table 4. Treatment duration was 8 to 12 weeks for >93% of patients across all racial groups. SVR defined as undetectable HCV RNA 12 weeks post-treatment was achieved in >95% of patients with similar rates across all racial groups (Table 4).

**Table 4:** Treatment regimens, durations, and outcomes by racial class.

	Non-Hispanic White (NHW)	Black/African American (BAA)	Hispanic White (HW)	Total
Viral load – week 4				
Undetectable	572 (87%)	201 (86%)	810 (89%)	1583 (86%)
Detectable	89 (13%)	32 (14%)	102 (11%)	223 (12%)
Viral load – post-treatment SVR				
Undetectable	684 (97%)	237 (94%)	919 (95%)	1840 (95%)
Detectable	25 (4%)	15 (6%)	50 (5%)	90 (5%)
Treatment regimens				
Harvoni +/− RBV	396 (43%)	183 (52%)	435 (36%)	1014 (50%)
Epclusa +/− RBV	290 (32%)	90 (26%)	404 (33%)	740 (30%)
Mavyret	163 (18%)	46 (13%)	275 (23%)	528 (21%)
Vosevi	35 (4%)	13 (4%)	53 (4%)	101 (4%)
Viekira +/− RBV	24 (3%)	13 (4%)	32 (3%)	69 (3%)
Other	8 (1%)	5 (2%)	15 (1%)	28 (1%)
Treatment duration				
8 weeks	151 (17%)	49 (15%)	205 (18%)	405 (20%)
12 weeks	665 (73%)	259 (79%)	824 (70%)	1748 (73%)
16 weeks	8 (1%)	0	11 (1%)	19 (1%)
24 weeks	71 (9%)	20 (6%)	133 (11%)	224 (10%)

SVR = sustained virologic response.

## 4. Discussion

HCV infection affects 71.1 million people worldwide and 2.4 million Americans chronically.^[11,12]^ According to the May 2016 World Health Organization (WHO) global action plan, the goal is to achieve elimination of HCV infection by 2030.^[13]^ However, only 20% of individuals with HCV infection know of their diagnosis and only 15% of those with known HCV infection have been treated. Additionally, higher incidence rates of HCV have been seen in non-Hispanic blacks and Hispanics.^[6,14]^ The major barriers to achieving these WHO global eradication goals are inadequate diagnosis and access to affordable point-of-care diagnostics and pan-genotypic DAA therapy.^[11]^ Given this background, we studied an almost 50% HW population in Texas in comparison to NHW and BAA to describe and report differences in demographic and socioeconomic characteristics amongst these populations.

In our cohort of patients in Texas, NHW patients were diagnosed with HCV an average of 6 to 9 years earlier than HW or BAA patients. This suggests important barriers to health care access particularly in primary care clinic screening to make a diagnosis. These barriers appear to affect largely the HW and BAA groups, which make up two-thirds of the population studied. Other prior studies have also shown low testing rates among racial and ethnic minorities.^[6]^ Although our findings are consistent with published data, our data is limited to patients in Texas and cannot be generalized to screening practices across the US.

In our analysis, all patients had access to treatment an average of 16 to 18 days after the decision was made to start treatment by the healthcare provider. Having Medicare or Medicaid coverage compared to private health insurance was previously reported to have a lower association with DAA initiation.^[9]^ Two-thirds of patients treated in this cohort had Medicare or Medicaid insurance compared to 37% being covered by private insurance. Despite this, time to initiation of DAA was similar in private, Medicare and Medicaid insured patients. A dedicated pharmacy team supervising prior authorization and treatment access such as in our patient population ensured 92% of patients were approved with first prior authorization request and consequently initiated DAA therapy in a timely fashion. Additionally, our specialized team tested all patients for HCV RNA at treatment week 4 to help assess whether patients were compliant with treatment. Those who had detectable HCV RNA at treatment week 4 underwent repeat HCV RNA testing at treatment week 8 and had a telehealth visit. This constant communication between healthcare team and patient can improve patient compliance and outcomes.

Patients in this cohort were treated between January 2017 and September 2021 and >95% achieved SVR yet 5% were unsuccessful. During that time, the preferred DAA regimens shifted. One factor associated with non-SVR is poor compliance. Some of the patients in this cohort were treated with older regimens that had a high pill burden. Over time, the HCV DAA regimens became less complicated for patients. Another advance was a transition to use of DAA regimens that had better pan-genotypic coverage regardless of presence or absence of cirrhosis.

In the US, the major hurdle has been linkage to care and access to DAAs for those of certain races and socioeconomic classes. Access to healthcare for these groups is often difficult so a specialized team including medical providers and pharmacists can overcome barriers. A pharmacist can work with the medical team to get DAA prior authorization in a timely fashion. The telehealth model with strong oversight by a medical team and pharmacist can continue the oversight to ensure compliance and safety. Taken together, the barriers once prohibiting individuals of certain races and socioeconomic classes can be minimized. The applicability of this model across large healthcare systems may be limited due to resource constraints.

Racial class or poor socioeconomic status did not impact a high SVR of >95% in our population. A limitation of this study is the fact that socioeconomic class is based on the patient’s home zip code. Patient’s income is not captured but location where they live can be correlated to their income. Historically, racial class has played a role in access to care for patients and in turn access to treatment, particularly access to older interferon-based treatments. Studies from the interferon era showed treatment rates were lower among Black patients than among White patients, even in settings with equal access to health care.^[9,15,16]^ This gap has been bridged by the newer DAAs with higher cure rates in patient groups such as BAAs, Hispanics and patient with human immunodeficiency virus co-infection.^[17]^ Given the high rates of SVR seen in our largely Hispanic cohort, one could extrapolate that robust patient education, close follow up through telehealth and good communication with the healthcare and dedicated pharmacy teams, may bridge important socioeconomic and racial barriers to treatment. A concerted effort on the part of healthcare personnel may be essential to achieving this goal.

## 5. Conclusion

In our study, BAA and HW were found to be equally eligible for HCV treatment compared to NHW which is contrary to what has been historically reported. Despite having more advanced disease and more negative socioeconomic factors, >94% of HW and BAA patients were cured. Continued patient education, communication with the healthcare team and a dedicated pharmacy team can lead to high adherence and >95% HCV cure rates regardless of race/ethnicity or underlying socioeconomic factors. This could prove to be crucial if WHO goals for eradication of HCV by 2030 are to be achieved.

## Acknowledgments

The authors would like to thank Ashley Neumann, Peggy Cabrera, Andrea Molina, Kathy Alderete, and Sarah Zuazua for assistance with data collection.

## Author contributions

**Conceptualization:** Lisa D. Pedicone, Fred Poordad.

**Data curation:** Eugenia Tsai, Jae Kim, Timothy Van Frank, Robert Mitchell, Ruben Ramirez, Richard Guerrero, Bryan Hanysak, Carmen Landaverde, Fabian Rodas, Eric Lawitz, Tamneet Basra, Kim Christensen, Clarissa Vaughn, Kim Hinojosa, Nina Olvera, Edna Caraballo-Gonzalez, Emma Pham, Fred Poordad.

**Formal analysis:** Saatchi Kuwelker, Lily Kuo, Tamneet Basra, Harry Nguyen, Lisa D. Pedicone, Fred Poordad.

**Investigation:** Eugenia Tsai, Jae Kim, Timothy Van Frank, Robert Mitchell, Ruben Ramirez, Richard Guerrero, Bryan Hanysak, Carmen Landaverde, Fabian Rodas, Eric Lawitz, Tamneet Basra, Harry Nguyen, Kim Christensen, Clarissa Vaughn, Kim Hinojosa, Nina Olvera, Edna Caraballo-Gonzalez, Emma Pham, Fred Poordad.

**Methodology:** Lisa D. Pedicone, Fred Poordad.

**Project administration:** Kim Christensen, Clarissa Vaughn, Lisa D. Pedicone.

**Supervision:** Eugenia Tsai, Kim Christensen, Emma Pham, Lisa D. Pedicone, Fred Poordad.

**Visualization:** Fred Poordad.

**Writing – original draft:** Saatchi Kuwelker, Lily Kuo, Lisa D. Pedicone.

**Writing – review & editing:** Saatchi Kuwelker, Eugenia Tsai, Lily Kuo, Jae Kim, Timothy Van Frank, Robert Mitchell, Ruben Ramirez, Richard Guerrero, Bryan Hanysak, Carmen Landaverde, Fabian Rodas, Eric Lawitz, Tamneet Basra, Harry Nguyen, Kim Christensen, Clarissa Vaughn, Kim Hinojosa, Nina Olvera, Edna Caraballo-Gonzalez, Emma Pham, Lisa D. Pedicone, Fred Poordad.
